# Are reporting guidelines used in infectious diseases publications? An analysis of more than 1,000 articles

**DOI:** 10.1017/ash.2023.492

**Published:** 2023-11-29

**Authors:** Aldo Barajas-Ochoa, Manuel Ramirez-Trejo, Aditee Dash, Jillian E. Raybould, Gonzalo Bearman

**Affiliations:** 1 Division of Infectious Diseases, Department of Medicine, Virginia Commonwealth University, Richmond, VA, USA; 2 Centro Universitario de Ciencias de la Salud, Universidad de Guadalajara, Guadalajara, Mexico

## Abstract

**Objective:**

To assess whether 16 reporting guidelines of Enhancing the QUAlity and Transparency Of Health Research (EQUATOR) were used in infectious diseases research publications.

**Design:**

This cross-sectional, audit-type study assessed articles published in five infectious diseases journals in 2019.

**Methods:**

All articles were manually reviewed to assess if a reporting guideline was advisable and searched for the names and acronyms of 16 reporting guidelines. An “advisable use rate” was calculated.

**Results:**

We reviewed 1,251 manuscripts across five infectious diseases journals. Guideline use was advisable for 973 (75%) articles. Reporting guidelines were used in 85 articles, 6.1% of total articles, and 8% (95% CI 6%–9%) of articles for which guidelines were advised. The advisable use rate ranged from 0.06 to 0.17 for any guideline, 0–0.08 for CONSORT, 0.53–1 for Preferred Reporting Items for Systematic Reviews and Meta-Analyses (PRISMA), and 0–0.66 for Transparent reporting of a multivariable prediction model for individual prognosis or diagnosis (TRIPOD) : The TRIPOD statement. No trends were observed across the five journals.

**Conclusions:**

The use of EQUATOR-related reporting guidelines is infrequent, despite journals and publishers promoting their usage. Whether this finding is attributable to knowledge, acceptance, or perceived usefulness of the guidelines still needs to be clarified.

## IntroductionTransparent Reporting of a multivariable prediction model for Individual Prognosis Or Diagnosis

The ultimate goal of publishing a scientific manuscript is to “supply information that helps scientists develop new hypotheses, and provide a foundation on which new scientific discoveries and inventions are built.”^1^ A research manuscript represents the “product” of the research process and the decision to publish it is based on multiple factors, which include the perceived impact the findings will have on advancement of the field. The manner in which a research manuscript is written is crucial in the determination to publish it. Effective manuscript writing requires the correct use of language together with accurate expression and sequencing of ideas to ensure that the expressed concepts, rationale, results, interpretation of results, and limitations are understood. These allow for the data to be used in replication and confirmation by others and in the development of further research.

For decades, there have been expressions of concern regarding the quality of reporting in research manuscripts.^2,3^ More recently, our group described how numerous research manuscripts with preventable deficiencies continue to be written and submitted^4^. To improve the reporting of research manuscripts, reporting guidelines that serve as structured tools for research report writing in health sciences have been developed. These reporting guidelines list the minimum necessary elements to increase the likelihood that most types of manuscripts can be reproduced by other researchers, can be used to support clinical decisions, or can be included in a systematic review. The EQUATOR (Enhancing the QUAlity and Transparency Of health Research) network global initiative develops and promotes these and other reporting guidelines^5^ (see Table 1). Examples of these include the CONSORT (Consolidated Standards of Reporting Trials; available since 1996) statement for the reporting of clinical trials, which most journals require to be followed in submitting the results of clinical trials for publication, as well as the STROBE (Strengthening the Reporting of Observational Studies in Epidemiology; available since 2007) and PRISMA (Preferred Reporting Items for Systematic Reviews and Meta-Analyses; available since 2009) guidelines for observational studies and systematic reviews, respectively.^5^

**Table 1.** Studied guidelines’ acronyms, full names, and websites.

Guideline acronym and full name	Website
**EQUATOR network**: Enhancing the QUAlity and Transparency Of health Research	https://www.equator-network.org/
**AGREE II**: Advancing guideline development, reporting, and evaluation in health care	https://www.equator-network.org/reporting-guidelines/the-agree-reporting-checklist-a-tool-to-improve-reporting-of-clinical-practice-guidelines/
**ARRIVE**: Animal Research: Reporting of In Vivo Experiments	https://www.equator-network.org/reporting-guidelines/improving-bioscience-research-reporting-the-arrive-guidelines-for-reporting-animal-research/
**CARE**: Consensus-based Clinical Case Reporting Guideline Development	https://www.equator-network.org/reporting-guidelines/care/
**CHEERS**: Consolidated Health Economic Evaluation Reporting Standards	https://www.equator-network.org/reporting-guidelines/cheers/
**CHERRIES**: Checklist for Reporting Results of Internet E-Surveys	https://www.equator-network.org/reporting-guidelines/improving-the-quality-of-web-surveys-the-checklist-for-reporting-results-of-internet-e-surveys-cherries/
**CONSORT**: CONsolidated Standards of Reporting Trials	https://www.equator-network.org/reporting-guidelines/consort/
**COREQ**: Consolidated criteria for reporting qualitative research	https://www.equator-network.org/reporting-guidelines/coreq/
**MOOSE**: Meta-analysis Of Observational Studies in Epidemiology	https://www.equator-network.org/reporting-guidelines/meta-analysis-of-observational-studies-in-epidemiology-a-proposal-for-reporting-meta-analysis-of-observational-studies-in-epidemiology-moose-group/
**PRISMA**: Preferred Reporting Items for Systematic Reviews and Meta-Analysis	https://www.equator-network.org/reporting-guidelines/prisma/
**RECORD**: REporting of studies Conducted using Observational Routinely-collected Data	https://www.equator-network.org/reporting-guidelines/record/
**SPIRIT**: Standard Protocol Items: Recommendations for Interventional Trials	https://www.equator-network.org/reporting-guidelines/spirit-2013-statement-defining-standard-protocol-items-for-clinical-trials/
**SQUIRE**: Standards for QUality Improvement Reporting Excellence.	https://www.equator-network.org/reporting-guidelines/squire/
**STARD**: Standards for Reporting Diagnostic accuracy studies	https://www.equator-network.org/reporting-guidelines/stard/
**STREGA**: STrengthening the REporting of Genetic Association Studies	https://www.equator-network.org/reporting-guidelines/strobe-strega/
**STROBE**: Strengthening the Reporting of Observational Studies in Epidemiology	https://www.equator-network.org/reporting-guidelines/strobe/
**TRIPOD**: Transparent reporting of a multivariable prediction model for individual prognosis or diagnosis	https://www.equator-network.org/reporting-guidelines/tripod-statement/

Publishers and several journals from different medical specialties, including infectious diseases, explicitly endorse the use of the guidelines cited above.^2,3,6^ Furthermore, there is evidence that following these guidelines can improve scientific manuscripts,^7–10^ although not in every case.^11^ Our group’s recent experience with a highly selective sample of rejected manuscripts^4^ and published manuscripts^12^ in another medical specialty indicates that the use of reporting guidelines is uncommon. Information regarding the use of reporting guidelines is scarce, and, to our knowledge, unavailable in the field of infectious diseases.

In this exploratory study, we evaluated the frequency and characteristics of the use of EQUATOR-related guidelines in research manuscripts published during 2019 by five high-impact infectious diseases journals.

## Methods

### Study design and eligibility

This is a cross-sectional study conducted between May and September 2022. We use the STROBE guidelines for reporting.^5^

We assessed original research manuscripts published in what we consider a typical year (2019) in five specialized journals in infectious diseases: *Journal of Infectious Diseases*, *Lancet HIV*, *Clinical Infectious Diseases*, *Journal of Infection*, and *Lancet Infectious Diseases*. Based on our previous work,^4,12^ five journals could be manually reviewed for approximately 900 articles, since each journal publishes approximately 180 original articles. The selected journals were a convenience sample according to the following criteria: that the journals focused on infectious diseases, and that, according to the indicators of Scimago Journal and Country Rank^13^ for 2019, they were within quartile 1 (ie, journals with the highest impact), had a high H index (ie, reflecting higher number of citations than publications) and that at least 20% of the articles published in 2019 were original research (to select journals that perform primary research).

### Article selection

All original research articles published in 2019 were selected from the website of each selected journal. The full articles for the selected titles were downloaded as portable document files (PDF) using an institutional account. One author (AB-O) verified that each PDF file was an original research article. Articles such as editorials, reviews, and letters were excluded as the EQUATOR-related guidelines are intended for reporting research.

### Assessment of the use of reporting guidelines

Two authors (MR-T and AB-O) used the Adobe search tool to search each PDF for words that indicated the use of a reporting guideline. We used the words “guideline,” “reporting guideline,” the guidelines’ acronyms (ie, EQUATOR, CONSORT, STROBE, PRISMA, TRIPOD, CARE, STREGA, ARRIVE, RECORD, MOOSE, SPIRIT, STARD, COREQ, AGREE, CHERRIES, CHEERS, and SQUIRE), and their corresponding full names (eg, Strengthening Reporting of Observational Studies in Epidemiology for STROBE and Consolidated Trial Reporting Standards for CONSORT; Table 1). All files in which one of these terms appeared was manually reviewed by one author (AB-O) to verify that a guideline was used; for each guideline, its use was marked as confirmed if the authors expressed use of the reporting guideline in any way.

### Advisability assessment of reporting guidelines

Whereas reporting guidelines exist for most types of research studies, there are some types of research article for which EQUATOR reporting guidelines have not been developed. Examples include in vitro experiments, mixed-design studies, and tissue sample analyses. One author (AB-O) manually reviewed the PDFs and followed the methods reported in a previous study by our group^12^ to assess whether the use of a reporting guideline was advisable and if so, suggested which guideline was relevant. Decisions on the advisable use of a guideline were made after reading the study design and methods and matching the study characteristics with a list including the names and definitions of all reporting guidelines. We then calculated the “advisable use rate,” defined as the number of articles per journal confirmed to have used a specific guideline divided by the number of articles per journal in which using a guideline was deemed advisable.

### Ethical considerations

This manuscript did not include clinical studies or patient data. Ethical approval and patient informed consent were not required.

### Statistical analysis

The results are reported using only descriptive statistics, given the present study design and low frequencies of the studied events. Differences in the number of articles among the studied journals precluded comparisons.

## Results

Overall, 2,291 articles were published during 2019 in the selected journals, of which we assessed all 1,251 (54%) research articles (Table 2). The use of a reporting guideline was deemed advisable for 937 (75%) articles. However, only 85 (6.1% of total articles) or 8% (95% confidence interval [CI]: 6% to 9%) of articles for which a guideline was deemed advisable used a reporting guideline.

**Table 2.** Frequency distribution of actual and advisable use of reporting guidelines and advisable use rate by selected ID-journals

	Lancet Infectious Diseases	Lancet HIV	Journal of Infectious Diseases	Journal of Infection	Clinical Infectious Diseases
Articles of all types published in 2019	447	196	581	185	882
Research articles to assess, n	107	45	386	106	607
Advisable articles for reporting guidelines, n (%)	88 (82)	40 (89)	247 (64)	80 (76)	482 (79)
Use of any reporting guidelines, n (AR*, %)	11 (12)	3 (7)	16 (6)	10 (12)	19 (3)
Reporting guidelines**	Actual use/advisable use (advisable rate*, %)				
CONSORT	1 /36 (2)	0/20 (0)	3/36 (8)	0/3 (0)	1/79 (1)
STROBE	3/30 (10)	0 /8 (0)	1/92 (1)	2/43 (4)	3/243 (1)
PRISMA or MOOSE	7/13 (53)	1/ 1 (100)	9/12 (75)	7/13 (53)	12/19 (63)
SPIRIT	0/0	0/ 0	0/0	0/0	0/3 (0)
STARD	0/1 (0)	0/0	2/2 (100)	0/10 (0)	1/9 (1)
TRIPOD	0/1 (0)	2/ 3 (66)	0/14 (0)	1/4 (25)	1/30 (3)
CARE	0/0 (0)	0/0	0 /1	0/0	0/3 (0)
STREGA	0/0 (0)	0/0	0/5	0/0	0/2 (0)
ARRIVE	0/0 (0)	0/0	1/60 (1)	0/0	0/0
RECORD	0/5 (0)	0/5 (0)	0/18 (0)	0/3 (0)	1/59 (1)
COREQ	0/0	0/0	0/0	0/0	0/2 (0)
AGREE	0/0	0 /1 (0)	0/5 (0)	0/1 (0)	0/13 (0)
CHERRIES	0/0	0/0	0/0	0/1 (0)	0/0
CHEERS	0/ 2 (0)	0/2 (0)	0/2 (0)	0/2 (0)	0/19 (0)
SQUIRE	0/0	0/0	0/0	0/0	0/1 (0)

* AR = advisable use rate; proportion related to the articles for which a guideline could have been used.

** Refer to Table 1 for the full names of the reporting guidelines.

Table 2 shows the frequency distribution for the use of specific reporting guidelines and the advisability use rate, by journal. Although the proportion of articles for which the use of a reporting guideline was deemed advisable ranged from 64% for *Journal of Infectious Diseases* to 89% for *Lancet HIV*, guideline use was low for all journals, with an actual use ranging from 3% for *Clinical Infectious Diseases* to 12% for both *Journal of Infection* and *Lancet Infectious Diseases* for those manuscripts for which guideline use was advisable. The STROBE guideline was found to be the most commonly advised, but its actual use for those advisable manuscripts across the five journals was 2%. Used for reporting clinical trials, the CONSORT guideline had an actual use of 3% of those advisable, and the PRISMA and MOOSE guidelines together had a 62% use rate of those advisable across the five journals. ARRIVE, the guideline for reporting animal research findings, and TRIPOD, which is used to report prediction models, were rarely used.

We found no mention of the AGREE, CHEERS, and CARE guidelines or the EQUATOR network. We did not observe identifiable trends in the studied variables across the five journals, and the low values for each variable precluded the assessment of associations.

## Discussion

In this exploratory study, we found that reporting guidelines were used in only a few (1 in 15) infectious diseases research manuscripts published during 2019 in five selected, quartile 1 infectious diseases journals. Low use rates were observed even in clinical trials (CONSORT) and animal research studies (ARRIVE), study designs that are typically subject to stricter regulations. This finding seems contradictory, considering that these guidelines are intended to help authors clearly communicate all the relevant information that a scientific report is expected to contain. Additionally, these guidelines are free for use, easily accessible, and customized to each study design; most journals and publishers endorse them, albeit to varying degrees.

We do not know the reasons for the infrequent use of reporting guidelines. It is unlikely that our findings were owing to errors in the assessment because the acronyms or full names of the guidelines would have appeared in the article text and/or reference list if they had been used. Alternatively, reporting guidelines may have been used but not mentioned by the authors of the published articles. However, this seems implausible because reporting that a guideline was followed increases the perceived robustness of the manuscript during peer review, so it is unlikely that authors would intentionally omit this information. Authors’ low awareness about reporting guidelines may be involved, but this is unlikely; CONSORT has been available for roughly 25 years, and most journals request this checklist to be followed when reporting clinical trials. Additionally, the EQUATOR network has been in operation for nearly 15 years,^5,14^ and three of the five journals we assessed clearly endorse its use in their instructions for authors’ section. Indeed, even after extensive distribution of the PRISMA statement in co-publications in multiple major medical journals during 2009,^15^ as well as the endorsement by major journals to use this guideline, we found that PRISMA and MOOSE guidelines for systematic reviews and meta-analyses were used in approximately half of the articles for which its use was advisable.

We are the first to assess the use of reporting guidelines in infectious diseases-focused journals, although a 2016 editorial published in the *Journal of Infection Prevention* endorses the use of reporting guidelines of the EQUATOR network and explains the rationale behind its decision^6^. Research in other medical fields also shows low use of reporting guidelines. In seven public health journals, only 1.5% of articles published between 2010 and 2013 included the acronyms CONSORT, PRISMA, or STROBE.^16^ A study in the field of urogynecology described that the CONSORT, PRISMA, and STROBE guidelines were explicitly mentioned in only 25% of clinical trials, 54% of systematic reviews, and 1.2% of observational studies, respectively.^17^ In a recent rheumatology study,^12^ our group found that reporting guidelines were used in 5.6% of total articles or in 7.2% (95% CI: 5% to 9%) of articles for which guidelines were advisable. Whereas differences between these findings and our results may be attributable to different methods and time-related effects, our conclusions are similar: reporting guidelines have been and continue to be infrequently used.

One characteristic that strengthens our study is the assessment of “advisability” for using a reporting guideline in each assessed manuscript. This study dimension provided a context for the number of manuscripts in which guidelines were actually used and the specific guidelines used. Nevertheless, our study has limitations that must be considered. First, although we included all 2019 original research publications from five selected infectious diseases journals, our sample does not represent the whole universe of infectious diseases journals and does not include noninfectious diseases-focused journals that publish research associated with the field. Second, the study’s exploratory, cross-sectional design, and low values obtained for the studied variables preclude causality assumptions or inferences. Third, the “advisable use” designation was assigned by a single individual with experience in bibliometrics by following a previously used but not validated method^12^; consequently, the possibility to over- or underestimate the advisable use is implicit. Despite this, our findings on reporting guideline use are consistent with those of other related publications. Fourth, we did not assess the degree to which manuscripts adhered to the reporting guideline recommendations when a reporting guideline was used; this assessment was beyond the scope of this study. However, several publications show that adherence to guidelines such as PRISMA and CONSORT has not been optimal in diverse areas, including rheumatology,^12^ cardiovascular medicine,^18^ pediatric urology,^19^ occupational health,^20^ obstetrics,^21^ anesthesiology,^22^ emergency medicine,^23^ internal medicine,^24^ head and neck cancer,^25^ and otorhinolaryngology.^26^ Finally, readers should be aware that the use of these reporting guidelines should not necessarily be equated to a high-quality presentation of study findings, but to ensuring the manuscript contains the necessary elements to properly evaluate the scientific endeavor and enhance reproducibility in science.

In summary, despite journal endorsement of their use, their free and easily accessible nature, and their potential benefits when used, reporting guidelines were used infrequently in research manuscripts published in five high-impact infectious diseases journals in this study. Although we cannot clarify the reasons behind this finding, our exploratory study is a starting point for future studies investigating issues such as whether authors are aware of existing guidelines, authors’ perception of the usefulness of known guidelines, and whether editors and publishers value existing guidelines. Further studies may also explore whether the use of reporting guidelines facilitates and save costs in the peer review process, which has been estimated to be 100 million hours in 2020 with an estimated monetary value of more than 1.5 billion USD based on time spent by reviewers in the United States,^27^ as well as whether the probability of manuscript acceptance increases with guideline use. We agree with the objective of the EQUATOR network, which is “to improve the reliability and value of published health research literature by promoting transparent and accurate reporting,”^5^ and we believe that the use of reporting guidelines can help in achieving that goal.

## Data Availability

The data underlying this article will be shared upon reasonable request to the corresponding author.
